# Fast and reliable identification of axons, axon initial segments and dendrites with local field potential recording

**DOI:** 10.3389/fncel.2015.00429

**Published:** 2015-10-27

**Authors:** Anders V. Petersen, Emil Ø. Johansen, Jean-François Perrier

**Affiliations:** Department of Neuroscience and Pharmacology, Faculty of Health and Medical Sciences, University of CopenhagenCopenhagen, Denmark

**Keywords:** axon initial segment, dendrite, modulation, spike triggered averaging, axon, plasticity

## Abstract

The axon initial segment (AIS) is an essential neuronal compartment. It is usually where action potentials are initiated. Recent studies demonstrated that the AIS is a plastic structure that can be regulated by neuronal activity and by the activation of metabotropic receptors. Studying the AIS in live tissue can be difficult because its identification is not always reliable. Here we provide a new technique allowing a fast and reliable identification of the AIS in live brain slice preparations. By simultaneous recording of extracellular local field potentials and whole-cell patch-clamp recording of neurons, we can detect sinks caused by inward currents flowing across the membrane. We determine the location of the AIS by comparing the timing of these events with the action potential. We demonstrate that this method allows the unequivocal identification of the AIS of different types of neurons from the brain.

## Introduction

The axon initial segment (AIS) is the gatekeeper of neurons. It is there that nerve impulses are initiated before propagating toward the terminal regions of the axon and back to the somato-dendritic compartments (Eccles, 1964; Stuart and Sakmann, 1994). During the past years, it has become evident that the AIS is not a rigid structure that only generates action potentials each time the membrane potential reaches a threshold value. The modulation of ion channels permeable for Na^+^, K^+^, or Ca^2+^ ions expressed in this compartment provide a high degree of plasticity. For example K_V_7.2 and K_V_7.3 ion channels produce a slowly activating persistent outward current at the AIS (Pan et al., 2006; Rasmussen et al., 2007). The activation of muscarinic receptors inhibits the current and thereby increases the firing frequency of neurons (Brown and Adams, 1980; Brown and Passmore, 2009). In cartwheel neurons in the dorsal cochlear nucleus, the activation of dopamine D_3_ receptors at the AIS specifically inhibits T-type calcium channels and thereby spike initiation (Bender et al., 2010). In spinal motoneurons, the activation of serotonergic 5-HT_1A_ receptors at the AIS inhibits the Na^+^ current responsible for the genesis of action potentials. This mechanism is responsible for the central component of motor fatigue occurring during prolonged efforts (Cotel et al., 2013; Perrier and Cotel, 2015).

**FIGURE 1:**
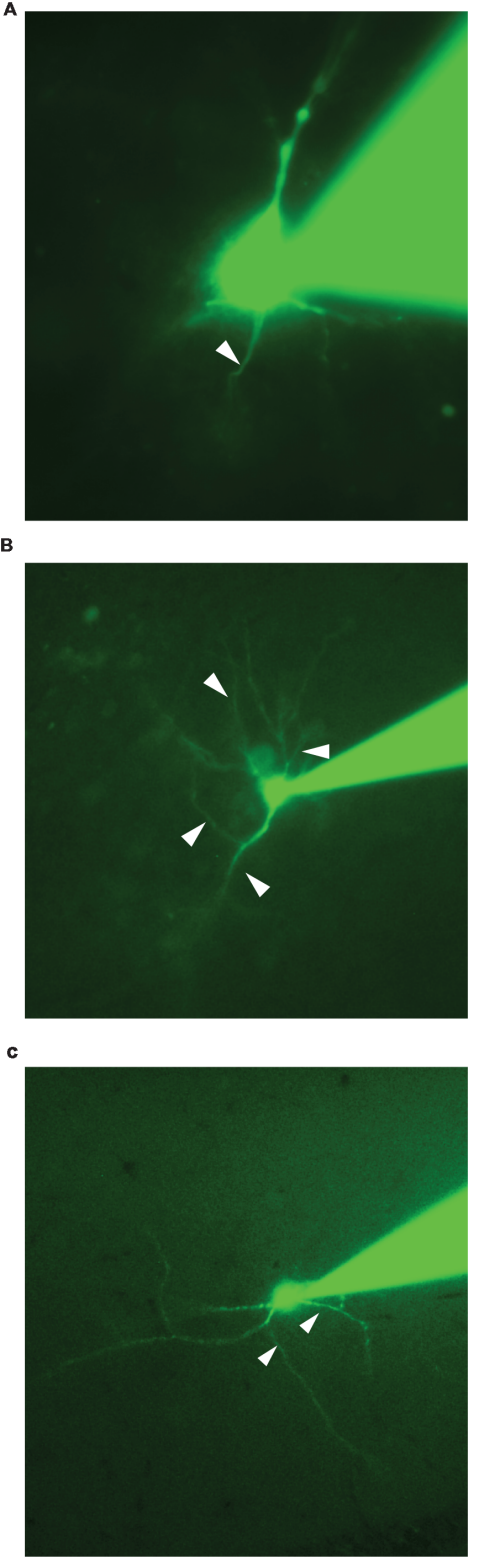
**Epifluorescence images of neurons obtained during their recordings. (A)** Pyramidal neuron from the neocortex. In the example one neurite is very likely to be the axon (arrow). **(B)** Neuron from the hippocampus. At least two neurites could correspond to the axon (arrows). **(C)** Interneuron located in the *stratum radiatum* of the CA1 region of the hippocampus. At least two neurites could correspond to the axon (arrows).

Identifying the AIS during an experiment is therefore highly relevant for physiological studies. However, this problem is far from being trivial because most neurons have several dendrites with diameters comparable to the ones of axons. For that reason, the visual identification of the axon during electrophysiological recordings can be equivocal (**Figure 1**). In addition, the axon sometimes derives from a first order dendrite rather from the soma (Ruigrok et al., 1984; Hounsgaard et al., 1988; Hausser et al., 1995; Thome et al., 2014). So far, the most reliable method for identifying axons requires multiple recordings with the patch-clamp technique. This has been successfully done for few types of neurons such as pyramidal cells from the neocortex (Stuart and Sakmann, 1994; Stuart et al., 1997). However, this procedure is difficult and only allows the recording of relatively thick or cut axons presenting a bleb (Kole et al., 2007). In cell cultures, it is possible to label the AIS of all neurons by means of GFP-tagged proteins specific for the AIS (Zhang and Bennett, 1998) or by means of a mouse monoclonal antibody recognizing an extracellular epitope of neurofascin (Schafer et al., 2009). However, this method has not been used in slices, probably due to the poor visibility of the tags. Another approach is to stain the axon by means of antibodies directed against specific markers such as ankyrin G (Hedstrom et al., 2008), tau protein (Binder et al., 1986), or sodium channels (Duflocq et al., 2008; Grubb and Burrone, 2010; Cotel et al., 2013). This method, being performed on fixed tissue after the end of the experiment, only allows testing if a given neurite was an axon or not, which is not optimal for studying the modulation of the AIS.

Here we provide a simple method based on extracellular field potential of neuronal processes combined with spike patch clamp recording of soma allowing fast and reliable identification of AIS in *in vitro* preparations.

## Materials and Methods

### Slice Preparation

After decapitation, the brain of C57BL/6 mice (Taconic) from P12 to P14 was removed and placed in cold artificial cerebrospinal fluid containing *N*-Methyl-D-glucamine 125 mM, KCl 2.5 mM, NaHCO3 26 mM, CaCl_2_ 2 mM, MgCl_2_ 1 mM, NaH_2_PO_4_ 1.25 mM, glucose 25 mM. Three hundred micrometer parasagittal slices from the brain were cut with a vibratome (VT1200; Leica Microsystems A/S, Germany). Slices were then incubated for and hour in a chamber containing oxygenated Ringer’s solution: NaCl 125 mM, KCl 2.5 mM, NaHCO3 26 mM, CaCl_2_ 2 mM, MgCl_2_ 1 mM, NaH_2_PO_4_ 1.25 mM, glucose 25 mM. Slices were then positioned in a recording chamber and continuously perfused with Ringer’s solution carbogenated by gassing with 95% O_2_ plus 5% CO_2_. Experiments were performed at room temperature. The surgical procedures complied with Danish legislation. This study was carried out in accordance with the recommendations of Department of Experimental Medicine of the University of Copenhagen. The protocol was approved by the Department of Experimental Medicine of the University of Copenhagen.

### Patch Clamp Recording

Visual guided patch clamp recording was performed with a Multiclamp 700B amplifier (Molecular Devices, USA). Neurons were visualized by means of a BW51WI microscope (Olympus, Japan) equipped with differential interference contrast. Patch-clamp electrodes were made of borosilicate glass pulled with a P-87 micropipette puller (Sutter Instruments; USA). They were filled with the following solution (in mM): 122 K-gluconate, 2.5 MgCl2, 0.0003 CaCl2, 5.6 Mg-gluconate, 5 K-HEPES, 5 H-HEPES, 5 Na2ATP, 1 EGTA, 2.5 biocytin, 0.01 Alexa 488 hydrazide, sodium salt (Life Technologies, USA), and KOH to adjust the pH to 7.4. Electrodes had a resistance ranging from 4 to 8 MΩ. Recordings were sampled at 100 kHz with a 16-bit analog-to-digital converter (DIGIDATA 1440; Molecular Devices, USA) and displayed by means of Clampex 10.2 software (Molecular Devices, USA). Neurons were isolated from their surrounding synaptic environment by blocking AMPA receptors with CNQX (20 μM, Tocris), NMDA receptors with AP5 (50 μM, Tocris) and GABA_A_ receptors with Gabazine (10 μM, Tocris).

### Local Field Potential Recording

Local field potential (LFP) electrodes were made with borosilicate capillaries pulled with a P-87 micropipette puller (Sutter Instruments; USA). They were filled with artificial cerebrospinal fluid of the same composition as detailed above. The LFP electrodes had a diameter of 1 μm and an input resistance of 4–7 MΩ. They were mounted on a 3-axis micromanipulator (Luigs and Neumann, Germany). The signal was recorded with a Multiclamp 700B amplifier (Molecular Devices, USA) and sampled at 100 KHz.

### Spike Triggered Average

The LFP electrode was positioned near the membrane of the recorded neuron. The acquisition of the signal was synchronized on the ascending phase of action potentials recorded with the patch-clamp technique. When the neuron was not firing spontaneously, positive bias currents were injected intracellularly. Between 200 and 2000 action potentials were acquired. Both signals were then averaged.

The spike threshold was determined as the first positive peak present on the third derivative of the voltage trace (Henze and Buzsaki, 2001). Extracellular events were considered only if their amplitude was more than five times the standard deviation of the baseline.

### Data Analysis

Data were analyzed by means of a custom program written in Matlab (Mathworks, Natick, USA) used to determine the time position of the spike threshold and to average extracellular recordings. The program is available at the following permalink: http://www.mathworks.com/matlabcentral/fileexchange/53161-axon-initial-segment-identifier.

## Results

We recorded twelve neurons from different brain regions including principal cells and interneurons from neocortex, midbrain and hippocampus. For some neurons, one neurite was an obvious candidate for being the axon. The neuron illustrated in **Figure 1A** is a pyramidal cell from the neocortex recorded in whole-cell configuration observed by means of fluorescence microscopy. In this example, the single neurite located between the basal dendrites is probably the axon (arrowhead). However, for other neurons, it was virtually impossible to determine which of the processes was the axon. In the example of **Figure 1B**, a pyramidal cell from the hippocampus has several processes leaving the soma in the basal region. One of them is probably an axon. However the similitude between diameters does not allow a clear distinction between the basal dendrites and the axon (arrowheads). Another example shows the morphology of an interneuron located in the *stratum radiatum* of the CA1 region of the hippocampus (**Figure 1C**). Here as well, it is difficult to know which of the processes corresponds to the axon. These examples demonstrate that the online identification of the AIS is far from being trivial. For that reason, we developed a method allowing a fast and reliable online identification of the AIS.

### Theoretical Basis for the Identification of the Axon Initial Segment

Any current being absorbed from the extracellular medium into a neuronal element appears as a sink (Nicholson and Freeman, 1975). The resulting lack of positive charges on the extracellular side generates a local negative electrical field potential. This occurs outside the AIS when action potentials are initiated and in the vicinity of other neuronal compartments that carry active propagation of electric signals. By comparing the timing of such field potentials with the action potential of a neuron, it should be possible to distinguish the AIS from other compartments.

**FIGURE 2:**
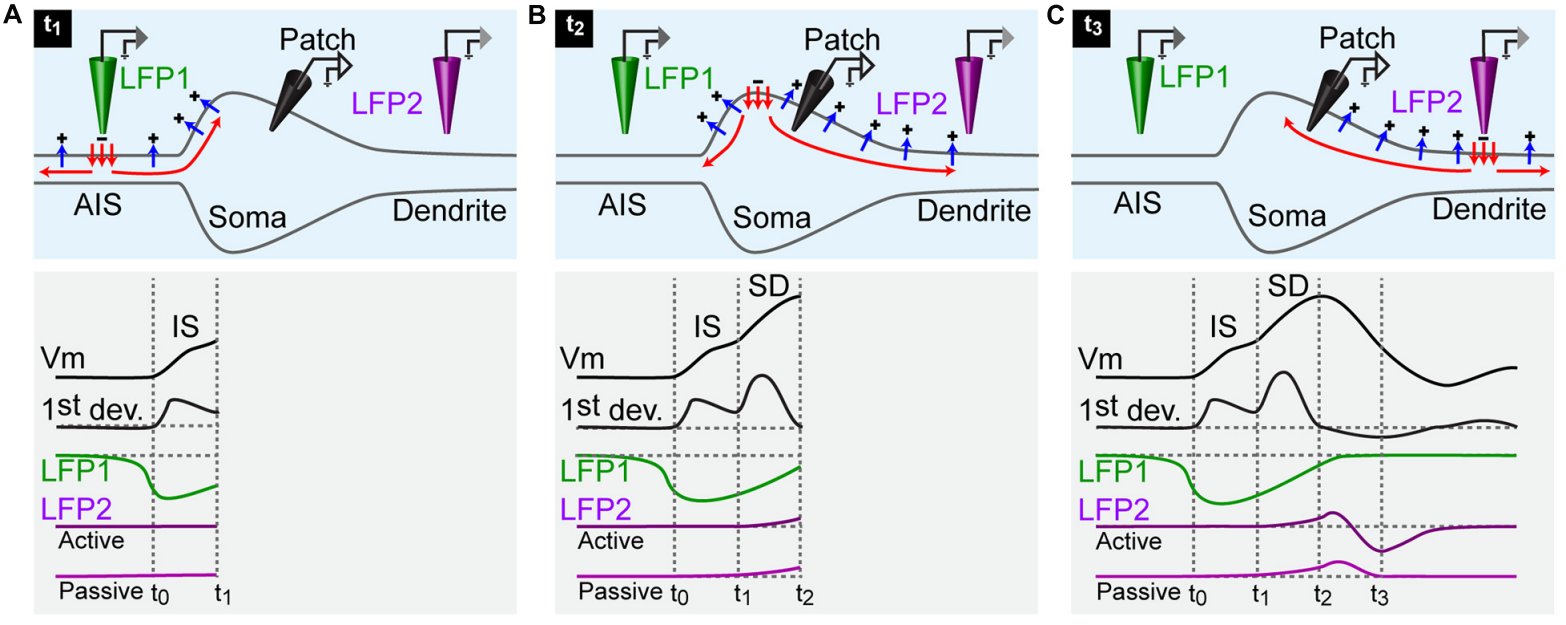
**Schematic representation of the currents responsible for an action potential.** For all panels: upper row, current flowing across the membrane of a neuron; lower rows: black traces, membrane potential of a neuron recorded by an intracellular electrode positioned in the soma and first derivative of the membrane potential; green trace: local field potential recorded by an extracellular electrode positioned near the AIS (LFP1); purple traces: local field potential recorded by an extracellular electrode positioned near an active and a passive dendrite (LFP2). **(A)** An action potential is generated at time 0 (t_0_). The inward current triggered by the activation of Na^+^ channels at the AIS spreads actively in the axon (red arrows) and leaks out passively through the membrane (blue arrows). The intracellular electrode measures a depolarization of the soma at time 1 (t_1_). The LFP electrode at the AIS detects a sink starting before the somatic depolarization. The dendritic LFP electrode does not record any change. **(B)** At time 2 (t_2_), the active current has invaded the whole soma. The membrane potential reaches the peak of the action potential. The LFP recorded at the AIS terminates while the LFP recorded in dendrites starts. **(C)** At time 3 (t_3_), the active current has reached the dendrite. The action potential recorded in the soma is finished. The dendritic LFP occurs as a positive deflection corresponding to the passive leakage of the current through the membrane. In case of an active dendrite, the positive event is immediately followed by a negative one caused by the presence of a sink.

The first event that occurs during an action potential is the activation of Na^+^ channels at the AIS (**Figure 2A**). This generates a negative field potential outside the AIS. The inward current then spreads passively in the cell, inducing a depolarization of the neighboring compartments. Because of the impedance mismatch, the resulting depolarization occurring in the soma is small (**Figure 2A**). Thus, the first component of an action potential recorded in the soma corresponds to the AIS spike (Eccles, 1964; Bean, 2007). This small depolarization, usually termed IS, is nevertheless sufficient to activate somatic Na^+^ channels, ensuring a regeneration of the inward current and the back propagation of the action potential in the soma and then in dendrites (SD component; **Figure 2B**). An inflection point is sometimes visible on the depolarizing phase of an action potential. For that reason, one can distinguish the IS from the SD component by plotting the first derivative of the voltage trace (Eccles, 1964; Bean, 2007), (**Figure 2**). After reaching the soma, the current spreads out passively into the dendrites and outside the cell through leak conductances (**Figure 2B**). A local field potential electrode located near a dendrite can therefore detect an excess of positive charges, characterized by a positive deflection occurring during the late phase of the action potential (**Figure 2C**). In case of active dendrites, the subsequent activation of voltage gated ion channels produces a sink following the positive deflection (**Figure 2C**). Thus, by comparing the timing of local field potentials with the one of action potentials recorded intracellularly, it is theoretically possible to determine if a given neurite is an axon or a dendrite. In addition, it allows figuring out if a dendrite is active or purely passive.

### Online Identification of the AIS

**FIGURE 3:**
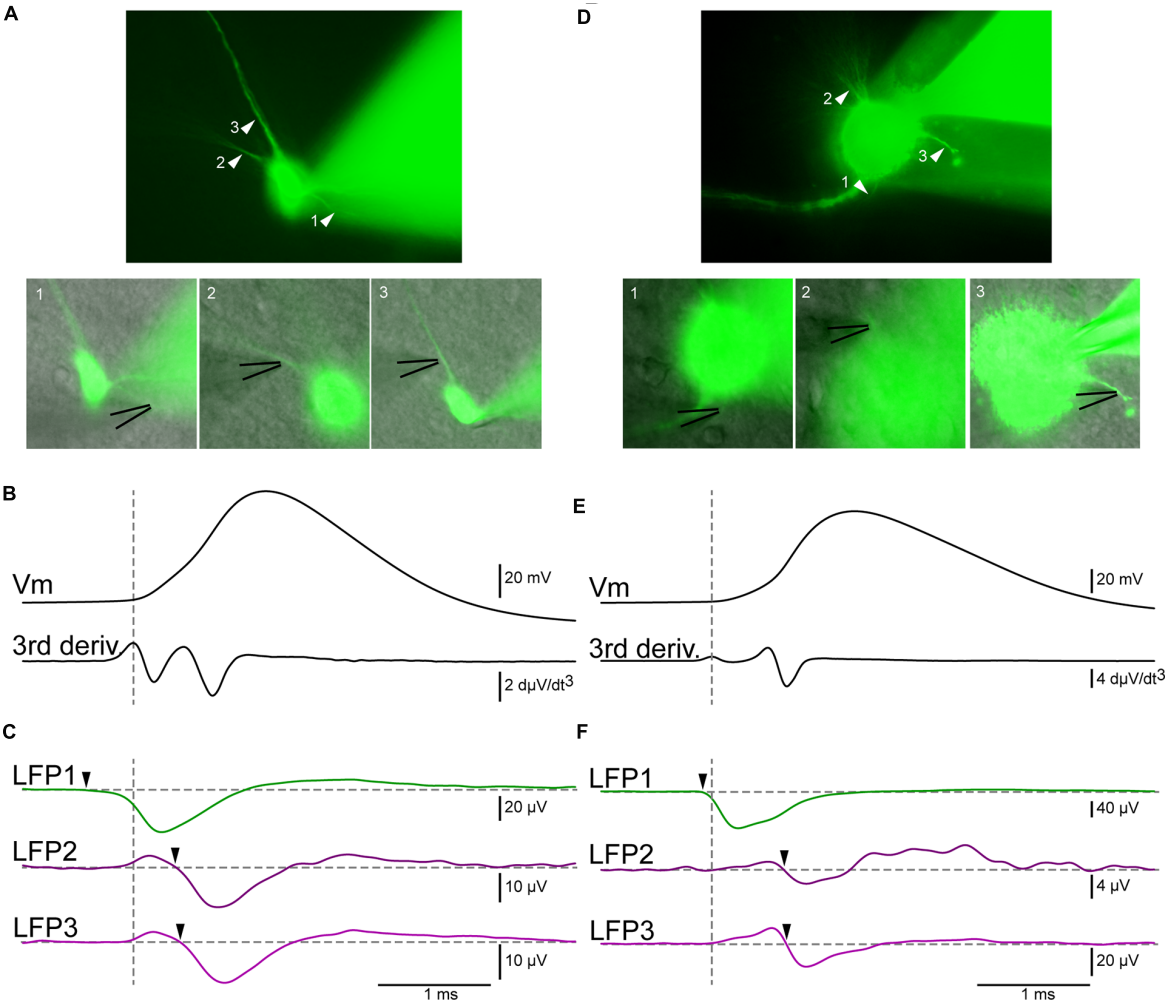
**LFP recordings allow the distinction of axon from dendrites. (A)** Epifluorescent pictures of a pyramidal cell from the hippocampus. The black lines indicate the positions of the LFP electrodes **(B)** Upper trace: membrane potential recorded by a patch electrode located at the soma. Lower trace: third derivative of the membrane potential. The maximum of the first positive peak corresponds to the beginning of the action potential (vertical dashed line). **(C)** Green trace: average of the LFP obtained at position 1 (2095 sweeps). A negative event (arrowhead) started before the action potential, indicating that the electrode was positioned near the AIS. Upper purple trace: Average of the LFP obtained at position 2 (1002 sweeps). Lower purple trace: average of the LFP obtained at position 3 (1063 sweeps). In both cases the LFP consisted of a positive and then a negative deflection (arrowhead) occurring after the beginning of the spike, demonstrating that the electrode was located near an active dendrite. **(D–F)** Neuron from the midbrain. **(D)** Epifluorescent pictures of the neuron. **(E)** Membrane potential and third derivative of the membrane potential. **(F)** Green trace: Average of the LFP obtained at position 1 (1101 sweeps). A negative event started before the action potential (arrowhead), indicating that the electrode was positioned near the AIS. Upper purple trace: average of the LFP obtained at position 2 (1003 sweeps). Lower purple trace: average of the LFP obtained at position 3 (1188 sweeps). Here again, a negative deflection (arrowhead) occurring after the beginning of the spike shows that the electrode was located near an active dendrite.

**FIGURE 4:**
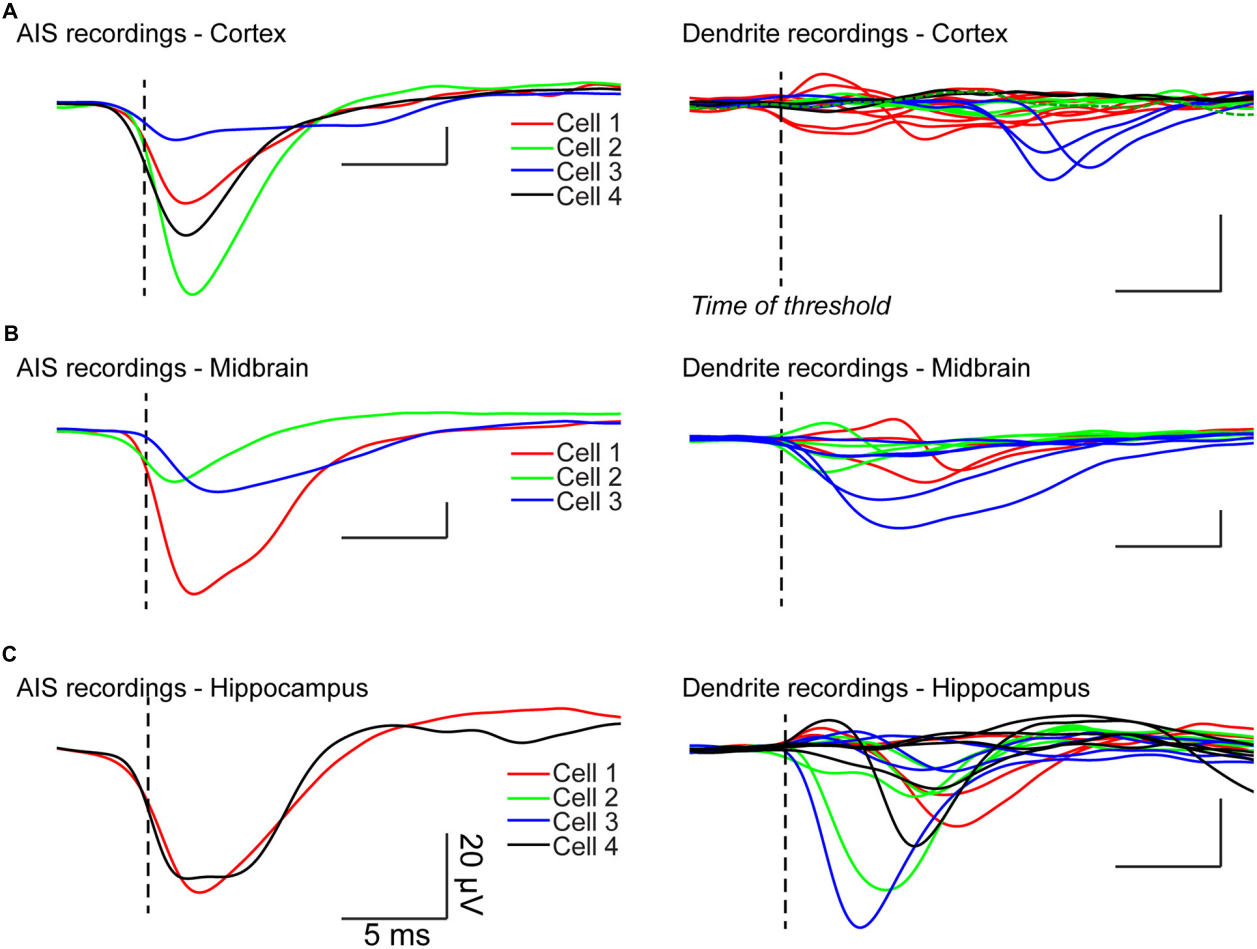
**LFP recordings from different neurons.** Left: LFP recordings obtained near AIS. Right: dendritic LFP recordings. **(A)** Cortical neurons. A passive dendrite belonging to cell 2 was recorded (dashed green line). **(B)** Midbrain neurons. **(C)** Hippocampal neurons.

We recorded the electrical activity of principal cells and interneurons from the hippocampus, neocortex, and midbrain. We visualized the somatodendritic arborisation of neurons by means of epifluorescence microscopy. We observed local field potential electrodes with bright field illumination and positioned it in the vicinity of different neuronal compartments by alternating fluorescence and bright field (**Figure 3A**). This procedure allows sub-μm precision. The distance between the LFP electrode and the membrane of the neuron tested was typically 1–3 μm and always less than 5 μm. We evoked action potentials by injecting intracellular positive bias currents. We found that single action potentials were usually not sufficient to induce events detectable from the background electrical noise. For that reason, we used the spike-triggered average technique. It consists in triggering the recording of the local field potential electrode on the ascending phase of the action potential. Each time the voltage trace crosses a given value (e.g., 0 mV), the recording starts. In order to analyze what happens just before the spike, we used a pre-trigger of 40 ms. After averaging 200–2000 spikes, the signal of the LFP electrode displayed clear events (**Figure 3C**). To determine if the extracellular electrode was positioned near the AIS, we compared the timing of the LFP events relative to the spike threshold calculated as the first positive peak present on the third derivative of the voltage trace, which provides a reliable estimation (Henze and Buzsaki, 2001; lower trace of **Figure 3B**; vertical dashed line). A negative event starting before the threshold measured at the soma indicated that the electrode was positioned near the AIS. In the example of **Figure 3C** obtained from a pyramidal neuron from the hippocampus, the LFP trace at position 1 (green trace in **Figure 3C**) displayed a negative event starting 400 μs before the spike threshold, suggesting that this position corresponded to the AIS. In contrast, the events detected at other positions started after the beginning of the spike. At positions 2 and 3 (purple traces in **Figure 3C**), the recording displayed a positive event followed by a negative event occurring 360–400 μs after the spike threshold. Because the negative event started after the start of the spike we concluded that the electrode was positioned near a dendrite. In addition, the presence of a negative peak indicated that the passive propagation of the signal was followed by an active one. This suggests that the backpropagation of the action potential was amplified by voltage-gated conductances. We tested the method for other types of neurons. **Figures 3D–F** illustrates the results obtained with a midbrain neuron for which the axon could not be visually identified with certainty. Here again, the LFP electrode detected a sink starting before the action potential at one position identified as the AIS (LFP1 in **Figure 3F**), and sources followed by delayed sinks at other positions (LFP2/3 in **Figure 3F**). **Figures 4A–C** illustrates all the average LFP recordings obtained at various positions near the membrane of eleven neurons. For 9/11 neurons, we could unambiguously distinguish the AIS from dendrites, demonstrating that the technique provided reliable results.

**FIGURE 5:**
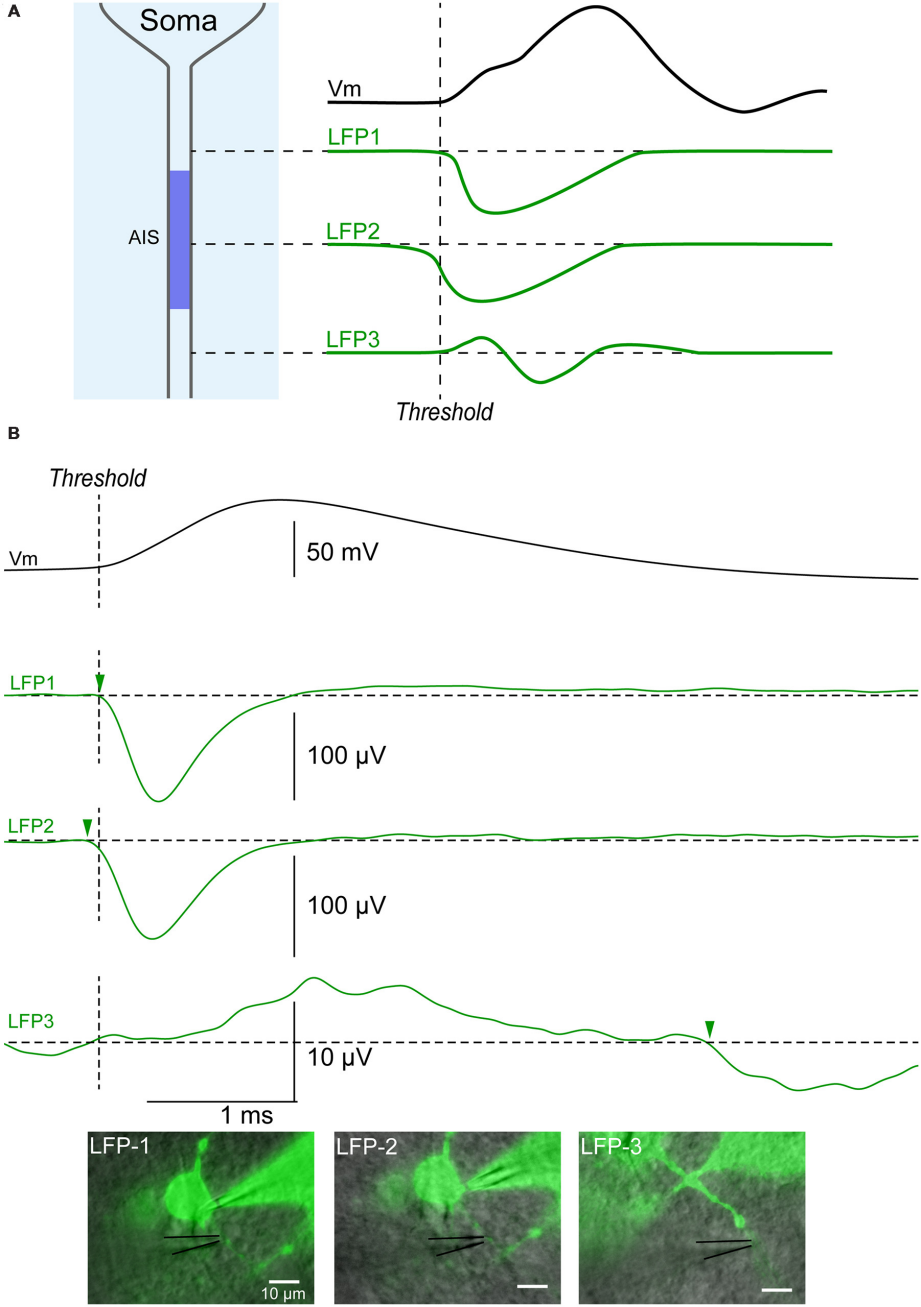
**Identification of the initial segment. (A)** Upper trace (black): action potential recorded intracellularly. Green traces: theoretical LFPs recorded along an axon. The first LFP occurring is a negative potential caused by the sink occurring at the AIS where the spike is generated (LFP2). An LFP recorded more proximal to the soma should be characterized by sink starting slightly later (LFP1), while an LFP more distal on the axon should start with a source followed by a sink (LFP3). **(B)** Example of LFPs recorded along the axon of a cortical neuron. Black upper trace: action potential recorded intracellularly. Green traces: LFPs. LFP1: LFP recorded 10 μm from the soma (515 sweeps). LFP2: LFP recorded 15 μm from the soma (287 sweeps). The latency for the negative event was the shortest at this position (arrowhead), suggesting that the action potential was initiated in this area. LFP3: LFP recorded 45 μm from the soma (1149 sweeps). A positive event suggesting the presence of a source was followed by a negative event (arrowhead) starting 4 ms after the beginning of the somatic action potential. Note the small amplitude of the signal when compared to LFPs recorded at positions 1 and 2.

We then tested if the initial segment could be localized more accurately along the axon by moving the electrode away from the soma. The LFP recording at the AIS should start earlier and have a bigger amplitude due to the higher density of voltage gated Na^+^ channels in this compartment (Kole et al., 2008), (**Figure 5A**). By contrast, recordings obtained more distally along the axon should appear as a positive deflection caused to the passive current preceding the spike and followed by a delayed negative deflection reflecting the inward current carried by Na^+^ ions (LFP3 in **Figure 5A**). In agreement, the LFP recorded along the axon of a cortical neuron was characterized by a negative event starting 50 μs before the spike threshold when positioned 15 μm from the soma of a cortical neuron (LFP2 in **Figure 5B**). When the LFP electrode was moved 5 μm closer to the soma, the negative event was detected 80 μs later (LFP1 in **Figure 5B**). When the extracellular electrode was moved 45 μm from the soma, along the axon, the LFP consisted of a positive event followed by a negative event starting 4 ms after the spike threshold (arrow in LFP3 in **Figure 5B**). It should be noticed that the amplitude of the recordings obtained at this position was one order of magnitude lower than the one obtained more proximally. These observations suggest that the spike was generated near position 2 (i.e., 15 μm from the soma). In agreement, position 2 was the only one identified as the AIS by our Matlab script (see Materials and Methods). Thus our technique does not only allow distinguishing dendrites from axon, but also permit determining the position of the AIS along an axon.

## Discussion

We have demonstrated a novel procedure allowing the online identification of the AIS during electrophysiological recording of neurons. Our method offers several advantages compared to others. First, it can be done in live tissue, while a neuron is recorded by means of the patch clamp technique. This facilitates the investigation of the physiological mechanisms involved in the modulation of the AIS. For example, one could focally apply agonists or antagonists by puffing or iontophoresing them from the LFP electrode, and determine if some receptors or ion channels are expressed at the AIS and if their activation has any impact on the excitability of the studied neuron (Perrier and Hounsgaard, 2003; Bender et al., 2010; Cotel et al., 2013). Second, the method is fast. The number of action potentials necessary for getting an acceptable signal to noise ratio with the LFP signal is about 200–1000. It is usually obtained within few minutes. By contrast, identifying the AIS by immunohistochemical staining with antibodies directed against proteins specific for the AIS requires several hours or days. Third, the technique is cheap. It only requires one extracellular recording electrode connected to an amplifier and does not necessitate any further investment. Fourth, the method is reliable provided that the patch recording is stable. It was possible to identify the axon for 10 of the 12 cells recorded in this study (i.e., more than 80%). Fifth, the method does not need any chemical that could potentially interfere with the physiological properties of the cell. Sixth, it is possible to determine the site of action potential initiation by mapping the LFP along the axon. Seventh, the size of the axon is not a limiting factor for the technique. Our approach allows the identification of the AIS of all neurons, independently of the diameter of the axon or of the presence of blebs caused by the slicing procedure.

The method we developed also has limitations. The identification of the AIS requires a good visibility of the cell, which is usually not the case for cells deeper than 80 μm from the surface of the slice. The method is invasive and the AIS has to be accessible with the LFP electrode. If the axon leaves the soma from below, or if it is positioned under the patch electrode, the method cannot be used. This is probably why we failed to identify two of the 12 AIS from the neurons of our sample. In addition, if the LFP is positioned near two close processes, it can be difficult to ascribe the signal to a particular one. In case of myelination, the signal recorded near axons may be difficult to record if the LFP electrode is not located near a node of Ranvier. Finally and importantly, the identification of the AIS relies on the fact that the spike is initiated in this compartment. This is not always the case. In mitral cells from the olfactory bulb and in some instances in pyramidal cells from the neocortex, the action potential can have a dendritic origin (Stuart and Sakmann, 1994; Chen et al., 1997; Schiller et al., 2000).

### Perspective

We believe that this new technique will prove useful for investigating the plasticity of the AIS. We tested our method in a slice preparation. It could also in principle be used *in vivo*, provided that one can visualize the whole somatodendritic arborisation of the investigated neuron, which would probably require multi-photon microscopy imaging techniques.

## Author Contributions

AP and J-FP, conception and design of research. AP, EJ and J-FP, performed experiments. AP analyzed data. AP and J-FP interpreted results of experiments. AP and J-FP wrote the manuscript. AP, EJ and J-FP approved final version of manuscript.

## Conflict of Interest Statement

The authors declare that the research was conducted in the absence of any commercial or financial relationships that could be construed as a potential conflict of interest.
